# Progranulin Inhibits Human T Lymphocyte Proliferation by Inducing the Formation of Regulatory T Lymphocytes

**DOI:** 10.1155/2017/7682083

**Published:** 2017-01-17

**Authors:** Kyu Hwan Kwack, Hyeon-Woo Lee

**Affiliations:** Institute of Oral Biology, School of Dentistry, Graduate School, Kyung Hee University, Seoul 02447, Republic of Korea

## Abstract

We have examined the effect of progranulin (PGRN) on human T cell proliferation and its underlying mechanism. We show that PGRN inhibits the PHA-induced multiplication of T lymphocytes. It increases the number of iTregs when T lymphocytes are activated by PHA but does not do so in the absence of PHA. PGRN-mediated inhibition of T lymphocyte proliferation, as well as the induction of iTregs, was completely reversed by a TGF-*β* inhibitor or a Treg inhibitor. PGRN induced TGF-*β* secretion in the presence of PHA whereas it did not in the absence of PHA. Our findings indicate that PGRN suppresses T lymphocyte proliferation by enhancing the formation of iTregs from activated T lymphocytes in response to TGF-*β*.

## 1. Introduction

Regulatory T lymphocytes (Tregs) play a pivotal role in preventing autoimmune reactions and regulating immune responses [1, 2]. Tregs are classified into two types. Naturally occurring Tregs (nTregs) are thymic in origin and suppress self-recognition T cells in secondary lymphoid tissues [3]. On the other hand, induced Tregs (iTregs) are formed in peripheral lymphoid tissues under the influence of TGF-*β* [4]. It has been reported that nTregs are generated to prevent autoimmune diseases, whereas iTregs are thought to control chronic inflammation [5]. Since iTregs are critical for modulating immune responses, understanding of how they are derived from naïve T lymphocytes and how they exert their effects is important because of their therapeutic potential [6–8].

Progranulin (PGRN) is a secreted glycoprotein that was first isolated from a teratoma prostate cancer cell line [9–11]. PGRN is a cysteine-rich protein that consists of 593 amino acids and can be converted into granulin (GRN) by extracellular proteases, including proteinase 3 and neutrophil elastase [12–14]. It is made up of 7.5 repeats of the GRN domain, and GRN was actually discovered before PGRN was cloned [14].

PGRN is widely expressed in cells such as hematopoietic cells, neurons, and immune cells such as macrophages, microglia, dendritic cells, and T lymphocytes [15]. PGRN is a growth factor and also promotes regeneration and tumorigenesis [16–18]. It plays important roles in wound healing [19], host defense, and inflammatory responses [20] and has neurotrophic properties [11, 21]. Full-length PGRN has an anti-inflammatory effect, while GRN is thought to have proinflammatory properties [14, 22]. Nevertheless, PGRN also has proinflammatory effects in specific diseases like obesity and insulin-resistant diabetes. In such cases, its proinflammatory effect is exerted by the production of IL-6 [23]. Therefore, the exact effect of PGRN varies depending on the pathological context.

The role of PGRN in the immune system is not clearly understood, although there is evidence that it is critical for modulating both innate and adaptive immunity [19, 24–27]. Thus, PGRN reduces TNF-*α*-induced IL-8 secretion [22] and binds to the tumor necrosis factor receptor (TNFR) blocking the TNF signal. Hence PGRN protects Tregs from negative regulation by TNF-*α* in humans [26]. Moreover, PGRN induces the formations of Tregs in mice [27]. However, exact inhibitory role of PGRN in human immune cells remains uncertain, and in the present work we examined its immune modulatory effects and their underlying mechanisms in human T lymphocytes.

## 2. Materials and Methods

### 2.1. Isolation and Culture of Human Peripheral Blood Mononuclear Cells

Peripheral blood was collected from healthy volunteers in accordance with the regulations of the Institutional Review Board of Kyung Hee Dental Hospital at Gangdong, and informed consent was obtained from all volunteers. Human Peripheral Blood Mononuclear Cells (hPBMCs) were fractionated from peripheral blood, using a Ficoll-Hypaque density gradient (1.077 g/ml, GE Healthcare, Piscataway, NJ, USA). CD3^+^ or CD4^+^ T lymphocytes were purified from the hPBMCs, using a magnetic MicroBead kit (Miltenyi Biotec, Bergisch Gladbach, Germany). Total hPBMCs were cultured with RPMI 1640 (Sigma Aldrich, St. Louis, MO, USA) supplemented with 10% FBS, 100 ug/ml streptomycin, and 100 U/ml penicillin. Isolated lymphocytes were cultured in RPMI 1640 medium containing 5% autologous serum, 100 ug/ml streptomycin, 100 U/ml penicillin, and 100 U/ml IL-2 (Peprotech, Rocky Hill, NJ, USA).

### 2.2. hPBMC Proliferation Assay

hPBMCs, CD4^+^, or CD3^+^ T lymphocytes (5 × 10^5^ cells/well) were treated with phytohemagglutinin (PHA, 12.5 ug/ml, Sigma Aldrich, St. Louis, MO, USA) to stimulate T lymphocyte proliferation in the absence or presence of PGRN and incubated in 48-well culture plates (Corning, NY, USA) for 5 days. EdU (5-ethynyl-2-deoxyuridine, 10 *μ*M, Invitrogen, Carlsbad, CA, USA) was added to the culture plates 18 hr before harvest. Based on a click reaction, EdU were reacted with azide, which is coupled to Alexa Flour 488 (Invitrogen, Carlsbad, CA, USA) dye. After harvesting the cells, the extent of cell proliferation was measured by flow cytometry (Beckman Coulter, Miami, FL, USA). Data were analyzed with FlowJo software (FlowJo, Ashland, OR, USA). Human TGF-*β* RI Kinase Inhibitor VI (SB431542, Selleckchem, Houston, TX, USA) or Treg inhibitor (Peptide P60, Abbiotec, San Diego, CA, USA) was used to block the formation of iTregs, and recombinant human TGF-*β* 1 protein (R&D System, Minneapolis, MN, USA) was used to induce the formation of iTregs.

### 2.3. TGF-*β* Assay

TGF-*β* concentrations in culture media were measured with a human TGF-*β*1 enzyme-linked immunosorbent assay (ELISA) kit (R&D Systems, Minneapolis, MN, USA). Plates were read on an iMark™ Microplate Absorbance Reader (Bio-Rad, Hercules, CA, USA) at 450 nm.

### 2.4. Staining of Regulatory T Cells

hPBMCs were treated with various concentrations of PGRN in the presence or absence of PHA. CD4^+^ T lymphocytes were stimulated with PHA plus PGRN in the presence or absence of Treg inhibitor or TGF-*β* RI Kinase inhibitor VI. In all experiments the cells were divided into two groups. One set was analyzed to assess cell proliferation and the other was stained with FITC-conjugated anti-CD4 antibody (eBioscience, San Diego, CA, USA) and PE-conjugated anti-Foxp3 antibody (eBioscience, San Diego, CA, USA). Flow cytometry was performed to determine the proportion of CD4^+^Foxp3^+^ Treg cells.

### 2.5. Statistical Analysis

All experiments were performed at least in triplicate, and the results are shown as means ± standard deviations. The statistical significance of differences was tested with GraphPad Prism 5 software (GraphPad software, La Jolla, CA, USA), using one-way ANOVA and Tukey's post hoc HSD test. *p* values less than 0.05 were considered statistically significant. Similar results were obtained in three to five independent experiments.

## 3. Results

### 3.1. PGRN Inhibits Human T Cell Proliferation

**Figure 1:**
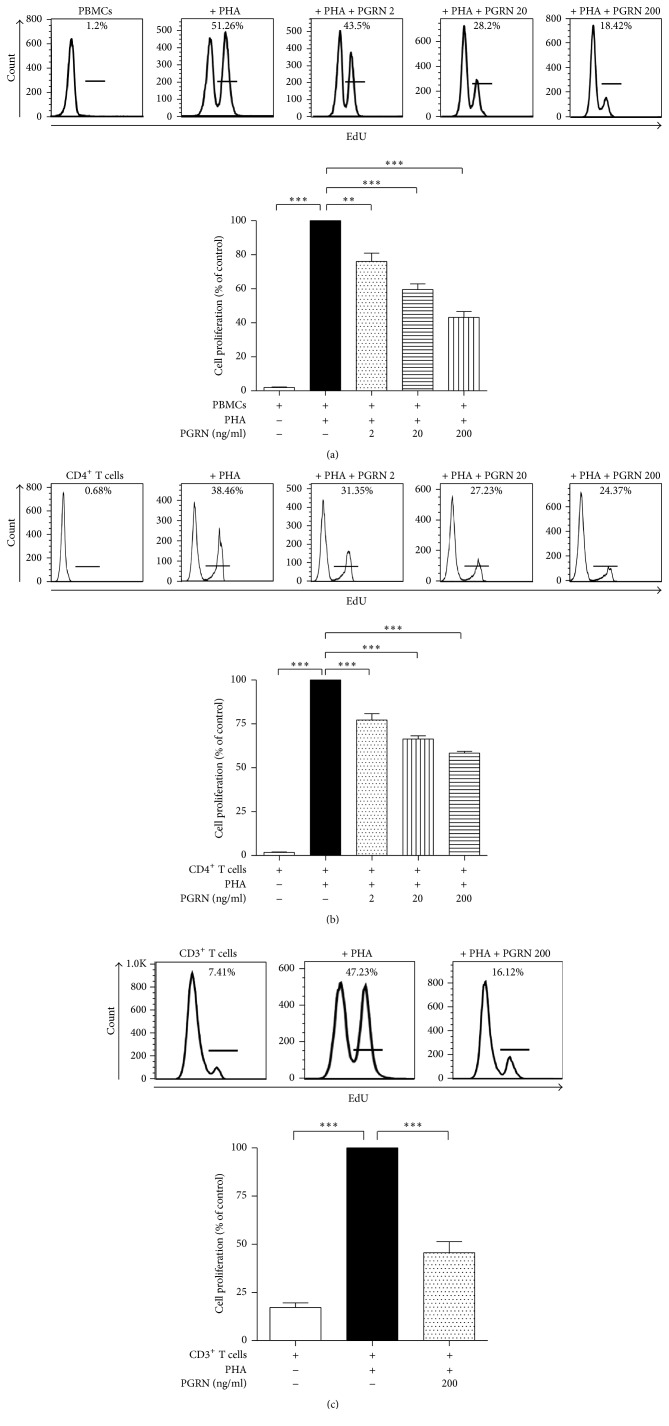
PGRN inhibits the proliferation of stimulated human PBMCs. hPBMCs were stimulated by PHA and incubated with PGRN at concentrations of 2, 20, and 200 ng/ml for 5 days (a). Isolated CD4^+^ T lymphocytes were incubated with PHA in the presence of PGRN at concentration of 2, 20, and 200 ng/ml for 5 days (b). Isolated CD3^+^ T lymphocytes were incubated with PHA in the presence of 200 ng/ml of PGRN (c). EdU was added to the culture medium 18 hr before cell harvest. Cells that had incorporated EdU were stained with Alexa Flour 488 and counted by flow cytometry. Data are presented as the means ± standard deviations of three experiments performed in triplicate. ^*∗∗*^*p* < 0.01, ^*∗∗∗*^*p* < 0.001. Similar results were obtained in four independent experiments.

To see whether PGRN suppresses T cell proliferation, hPBMCs were treated with PHA in the presence or absence of PGRN. As shown in Figure 1(a), PGRN inhibited T cell proliferation in a concentration-dependent manner. To confirm that this inhibition occurs in both total T cells and CD4^+^ T cells, we purified CD3^+^ or CD4^+^ T lymphocytes and stimulated them with PHA in the absence or presence of PGRN. As shown in Figures 1(b) and 1(c), PGRN reduced the proliferation of CD3^+^ and CD4^+^ T lymphocytes.

### 3.2. PGRN Increases the Number of iTregs

**Figure 2:**
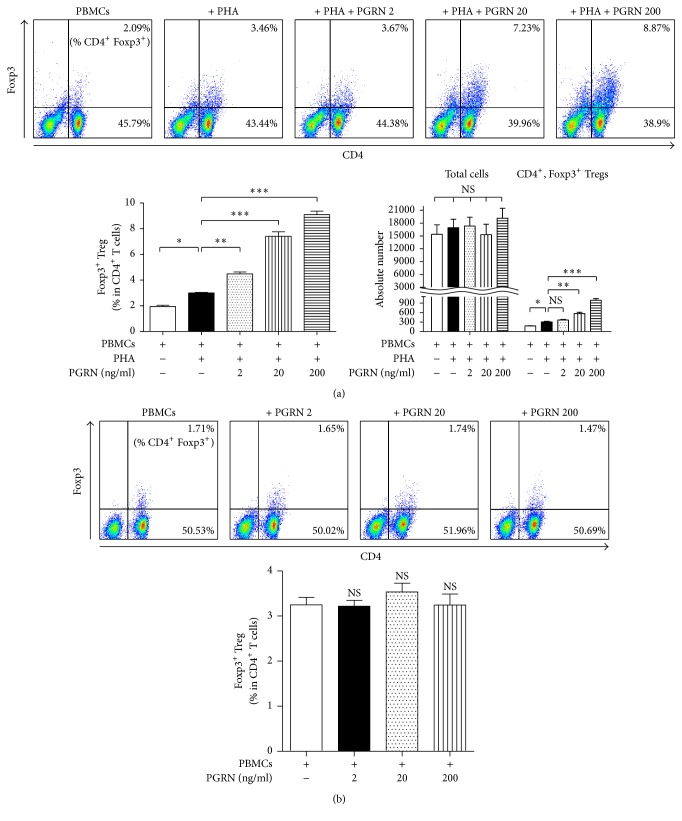
PGRN enhances numbers of CD4^+^Foxp3^+^ Tregs under conditions of stimulation. hPBMCs were incubated with PHA plus PGRN at concentrations of 2, 20, and 200 ng/ml for 5 days (a). hPBMCs were cocultured with PGRN at concentrations of 2, 20, and 200 ng/ml for 5 days (b). Cells were collected and stained as described in Section 2. Stained cells were analyzed by flow cytometry. In (a), left bar graphs show the percentage of CD4^+^Foxp3^+^ iTreg in CD4^+^ T lymphocytes. Right bar graphs indicate cell numbers of total cells and CD4^+^Foxp3^+^ iTreg, respectively. Data are presented as the means ± standard deviations of three experiments performed in triplicate. ^*∗*^*p* < 0.05, ^*∗∗*^*p* < 0.01, ^*∗∗∗*^*p* < 0.001, or ns (not significant). Similar results were obtained in five independent experiments.

To test whether PGRN promotes the production of Tregs as reported in mice [27], we examined its effect on numbers of iTregs produced from activated hPBMC in response to PHA. PGRN increased the number of CD4^+^Foxp3^+^ iTregs in a concentration-dependent manner, but it did not induce the frequency of iTreg in the whole CD4^+^ T population (Figure 2(a)). It is noteworthy that PGRN had no effect on iTreg formation from hPBMCs in the absence of PHA (Figure 2(b)).

### 3.3. PGRN Suppresses the Proliferation of T Lymphocytes through iTregs

**Figure 3:**
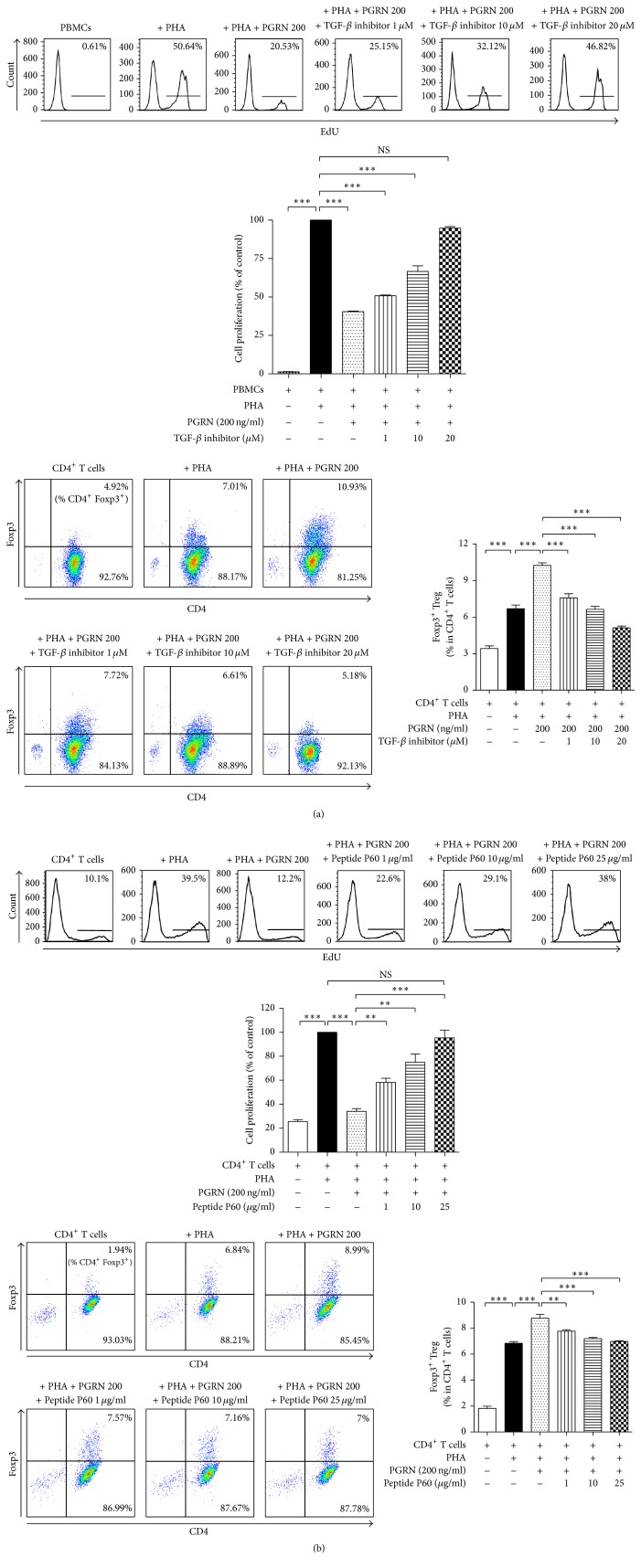
PGRN exerts its inhibitory effect by inducing the conversion of CD4^+^ T lymphocytes to iTregs via TGF-*β*. CD4^+^ T lymphocytes were stimulated with PHA and incubated with PGRN in the presence or absence of 1, 10, and 20 *μ*M of TGF-*β* inhibitor for 5 days (a). CD4^+^ T lymphocytes were stimulated with PHA and incubated with PGRN in the presence or absence of 1, 10, and 25 *μ*g/ml of Treg inhibitor for 5 days (b). Treated cells were collected and divided into two; one lot was stained for iTregs and the other was analyzed by cell proliferation assay. Flow cytometry was performed to determine iTregs and the proportion of iTregs. Upper panels in show the percentage of CD4^+^ T lymphocytes incorporated with EdU. Lower panels show the percentage of CD4^+^Foxp3^+^ iTreg ((a) and (b)). Data are presented as means ± standard deviations of three experiments performed in triplicate. ^*∗∗*^*p* < 0.01, ^*∗∗∗*^*p* < 0.001, or ns (not significant). Similar results were obtained in three independent experiments.

**Figure 4:**
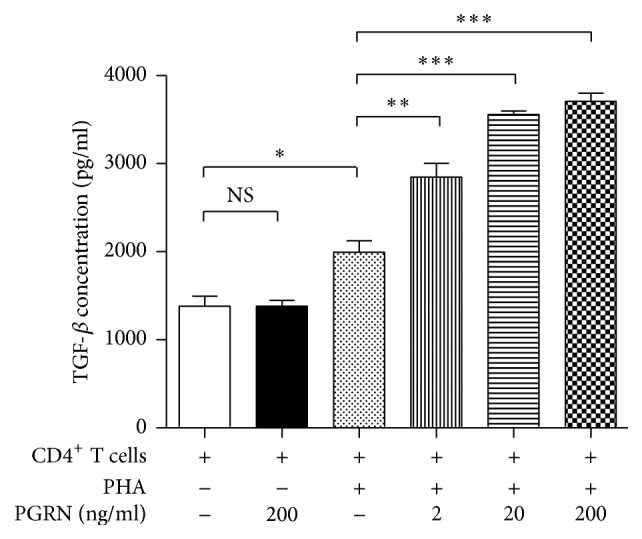
PGRN drives TGF-*β* secretion under the conditions of stimulation. CD4^+^ T lymphocytes were incubated with or without PHA in the presence or absence of indicated concentrations of PGRN for 5 days. TGF-*β* levels in the supernatants were measured by ELISA. Data are the means ± standard deviations of three experiments performed in triplicate. ^*∗*^*p* < 0.05, ^*∗∗*^*p* < 0.01, ^*∗∗∗*^*p* < 0.001, or ns (not significant). Similar results were obtained in four independent experiments.

**Figure 5:**
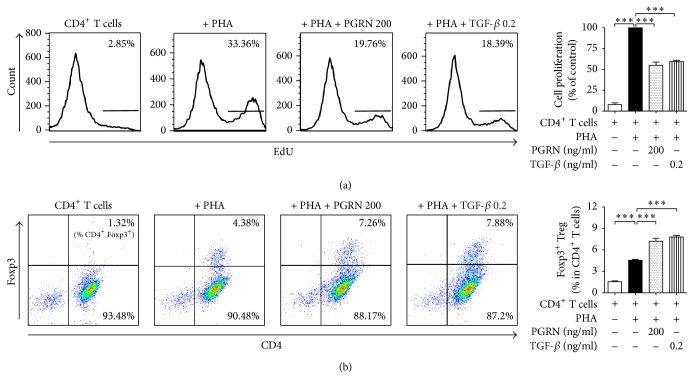
TGF-*β* inhibits CD4^+^ T lymphocyte proliferation and induces iTreg formation as well as PGRN. CD4^+^ T lymphocytes were incubated with PHA in the presence of 200 ng/ml of PGRN or 0.2 ng/ml of TGF-*β* for 5 days. For the proliferation assay, cells that had incorporated EdU were counted by flow cytometry and Foxp3^+^ T lymphocytes among the CD4^+^ T lymphocytes were analyzed by flow cytometry. (a) shows the percentage of CD4^+^ T lymphocytes incorporated with EdU. (b) shows the percentage of CD4^+^Foxp3^+^ iTreg. Data are the means ± standard deviations of three experiments performed in triplicate. ^*∗∗∗*^*p* < 0.001. Similar results were obtained in four independent experiments.

To examine whether the iTreg formation stimulated by PGRN was responsible for the inhibition of T cell proliferation, we incubated CD4^+^ T lymphocytes with PHA plus PGRN in the presence or absence of various concentrations of a Treg inhibitor or a TGF-*β* inhibitor. Peptide P60, a Treg inhibitor, and the TGF-*β* inhibitor almost completely reversed the PGRN-induced inhibition of CD4^+^ T cell proliferation as well as Treg formation (Figures 3(a) and 3(b)). This indicates that the iTregs induced by PGRN suppress PHA-mediated CD4^+^ T cell proliferation. Our data also suggest that PGRN stimulates the formation of iTregs from activated CD4^+^ T cells. To confirm that PGRN enhances TGF-*β* secretion, we incubated CD4^+^ cells with or without PGRN in the presence or absence of PHA and measured TGF-*β* levels in the culture media. As shown in Figure 4, PGRN-induced TGF-*β* secretion in the presence of PHA whereas it did not in the absence of PHA. These data indicated that CD4^+^ cell stimulation was prerequisite for PGRN-mediated TGF-*β* secretion. In Figure 5, incubating CD4^+^ T cells with TGF-*β* inhibited CD4^+^ cell proliferation and induced iTreg formation as comparable as with PGRN.

## 4. Discussion

In this work we first demonstrated that PGRN suppressed the proliferation of human T lymphocytes by enhancing their conversion into iTregs and went on to show that inhibition of iTreg formation with Peptide P60 completely reversed the immunosuppressive effect of PGRN. Moreover blocking TGF-*β* signaling, which is pivotal for iTreg differentiation, also completely abolished the effect of PGRN on T lymphocytes. Also, PGRN did not promote iTreg formation from T lymphocytes that were not incubated with PHA. This indicates that PGRN does not act on naïve T cells or on fully differentiated Tregs. Based on these results, we conclude that PGRN acts on activated T lymphocytes to enhance their conversion into iTregs rather than on already-differentiated iTregs. This idea is supported by a previous study showing that PGRN promoted the TGF-*β*-mediated formation of mouse iTregs [27].

PGRN-binding proteins have been studied in different tissues under various conditions. Sortilin has been reported as a binding protein for PGRN. The interaction between sortilin and PGRN is thought to be crucial to regulate PGRN trafficking in neurons [28, 29]. In addition, Zhou et al. found that prosaposin interacted with PGRN and facilitated sortilin-independent PGRN trafficking via the cation-independent mannose 6-phosphate receptor [30].

Moreover, it has been reported that PGRN binds to TNF receptors [26]. Although Chen et al. failed to demonstrate these interactions in their assay, recent studies have provided independent evidences that confirm the interactions of PGRN with TNFR in various cell types, including human lymphocytes [31–34].

Therefore, the identity of the target of PGRN and how it acts remain to be uncovered. Although we do not know the exact mechanism(s) by which PGRN promotes iTreg formation, we suggest the following model: (1) the PGRN receptor (PGRNR) is expressed on T lymphocytes only when they are activated or on iTregs induced by PHA; (2) PGRN/PGRNR-mediated intracellular signaling increases TGF-*β* expression and secretion; (3) this, in turn, induces Treg formation. Identification of the PGRNR and its intracellular signal transduction pathway will be critical for understanding its immunosuppressive action.

In conclusion, we have shown that PGRN has the ability to inhibit the proliferation of human CD4^+^ T cells by inducing them to differentiate into iTregs as a result of TGF-*β* production. Our findings suggest that PGRN might be used to treat autoimmune diseases or chronic inflammatory disease or to facilitate allogeneic stem cell transplantation.
